# Supplementary figures and images for: Clusters of deep intronic RbFox motifs embedded in large assembly of splicing regulators sequences regulate alternative splicing
Source: PLoS Genet. 2025 Sep 9;21(9):e1011855. doi: 10.1371/journal.pgen.1011855 (PMC12435732; doi:10.1371/journal.pgen.1011855)

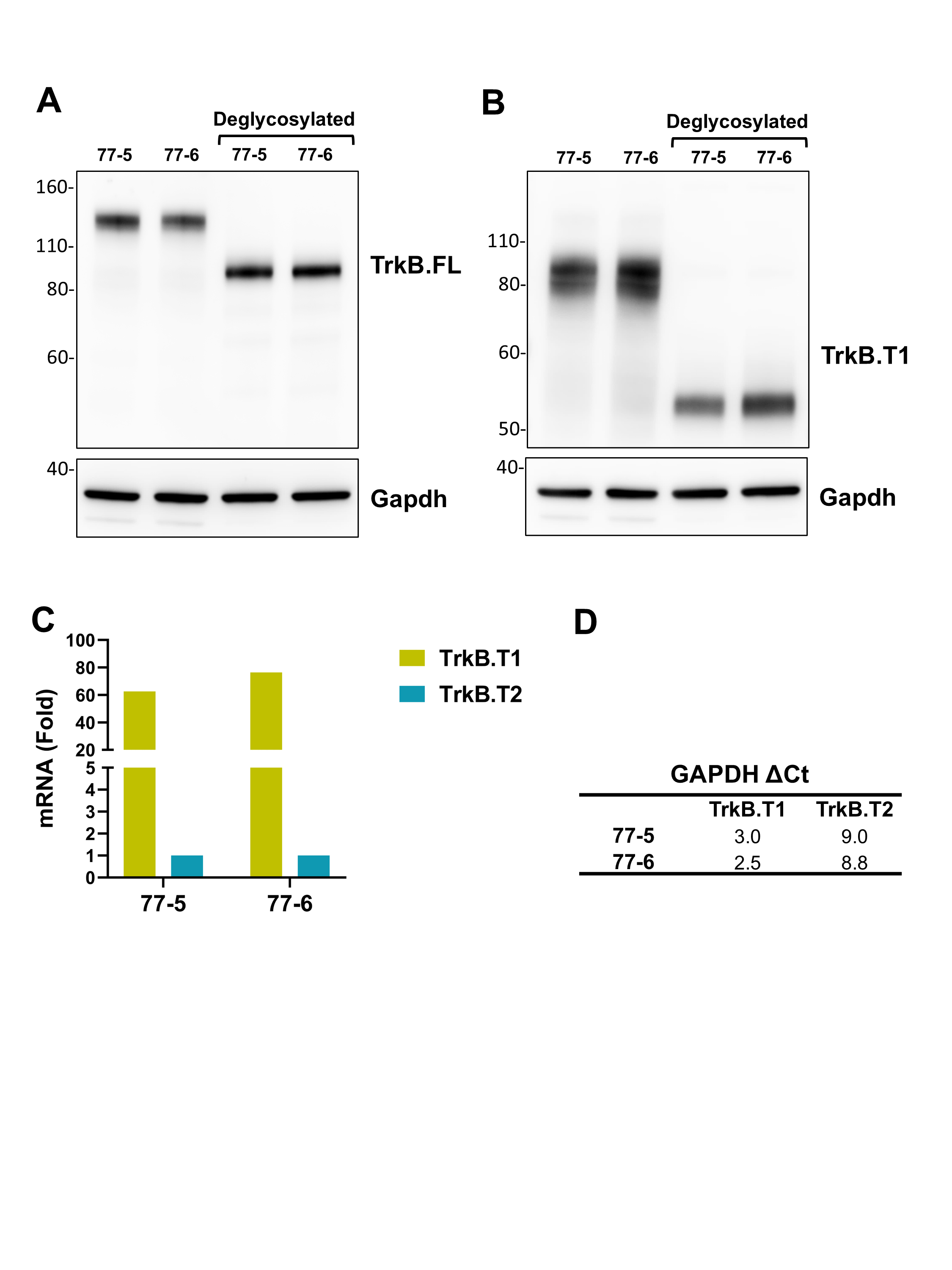

Supplement: S1 Fig — FL (A) or TrkB.T1 (B). Antibodies against Gapdh were used as a loading control. Note the significant decrease in size of both TrkB.FL and TrkB.T1 bands after deglycosylation and the presence of a single band following deglycosylation. (C, D) RT-PCR analysis of the same clones as in A, B analyzed for truncated TrkB.T1 and TrkB.T2 expression. Note the almost negligeable expression of TrkB.T2 relative to TrkB.T1 further confirming that TrkB.T1 is the main TrkB truncated isoform expressed by the BAC. (TIF) [file pgen.1011855.s001.tif]

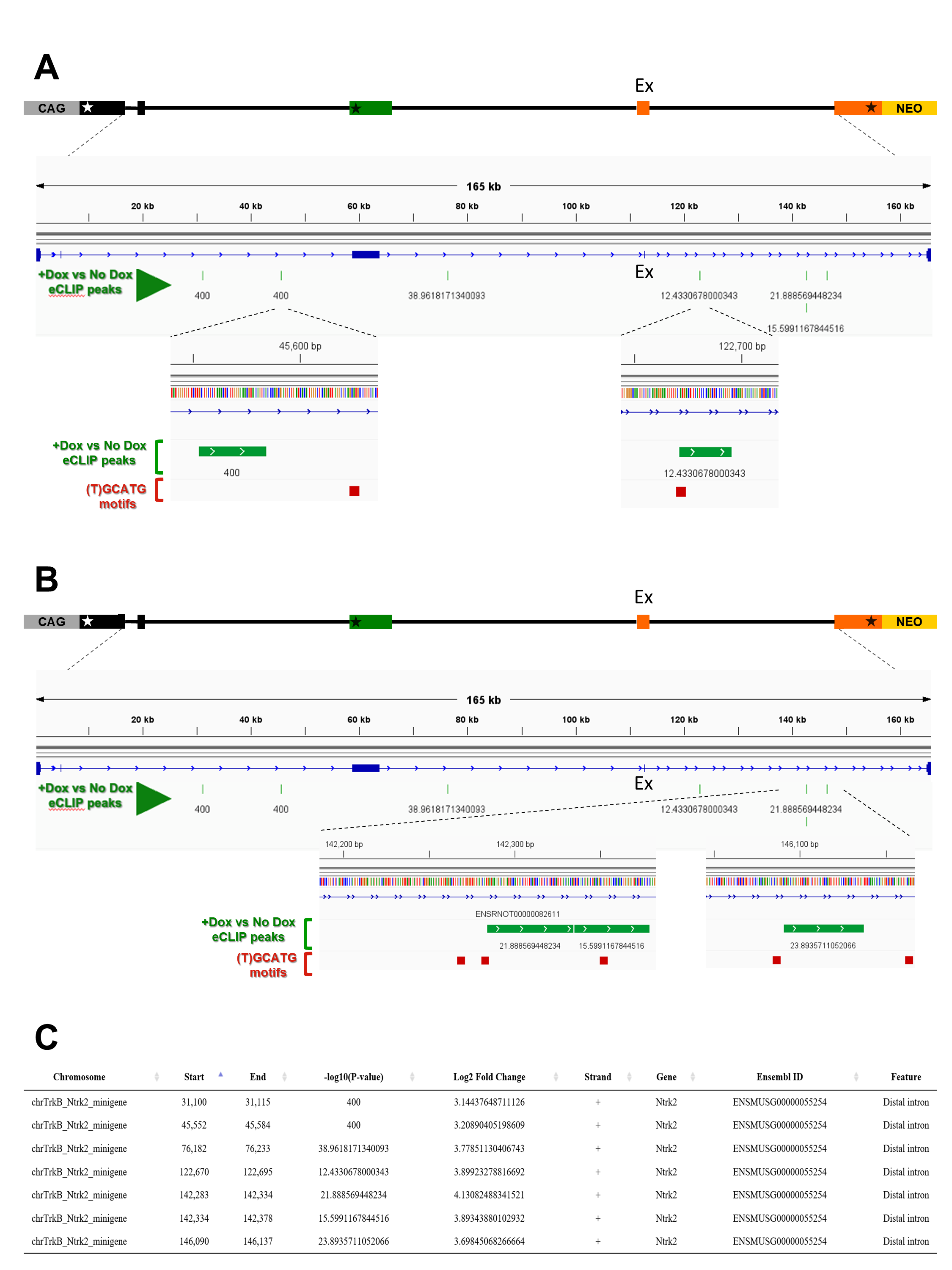

Supplement: S2 Fig — eCLIP peaks were derived by subtracting the signal obtained in the absence of RbFox1 (-Dox), considered as background, from the signal from the same cells (line 77−5 from Fig 1) expressing RbFox1. In green are seven statistically significant eCLIP peaks, all in distal intronic regions. Numbers under each eCLIP peak indicate the p-value (-log10). Below are the enlargements of the areas containing the eCLIP peaks (green) not depicted in Fig 2 relative to the position of RbFox1 (T)GCATG binding motifs (red). (C) Location, statistics, and fold change enrichment of the seven eCLIP peaks found across the minigene sequence shown in Fig 2. (TIF) [file pgen.1011855.s002.tif]

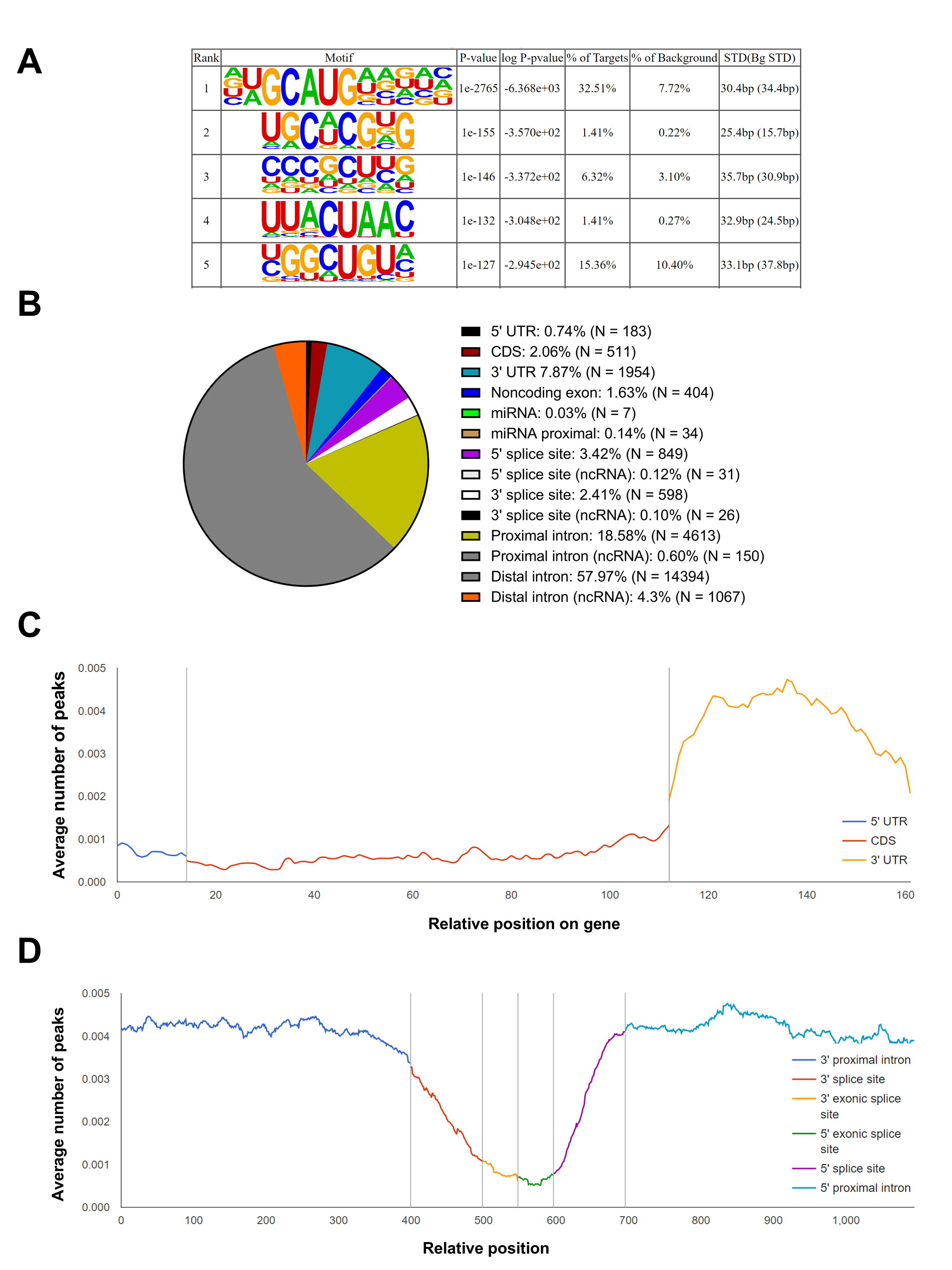

Supplement: S3 Fig — (A) Top five enriched motifs identified in CLIP-seq peaks by the HOMER motif analysis. (B) Pie chart depicting the relative frequency of eCLIP peaks that map to each specific gene region (with a peak Log2 fold enrichment ≥ 3 and p-value ≤ 0.001). (C, D) Peak Metagene Plot, depicting the average number of peaks mapped to the specific genomic regions indicated in B. The number of peaks was calculated for each gene region followed by normalization with the length of the regions. The average number of peaks was then calculated for a set number of positions along the regions. (TIF) [file pgen.1011855.s003.tif]

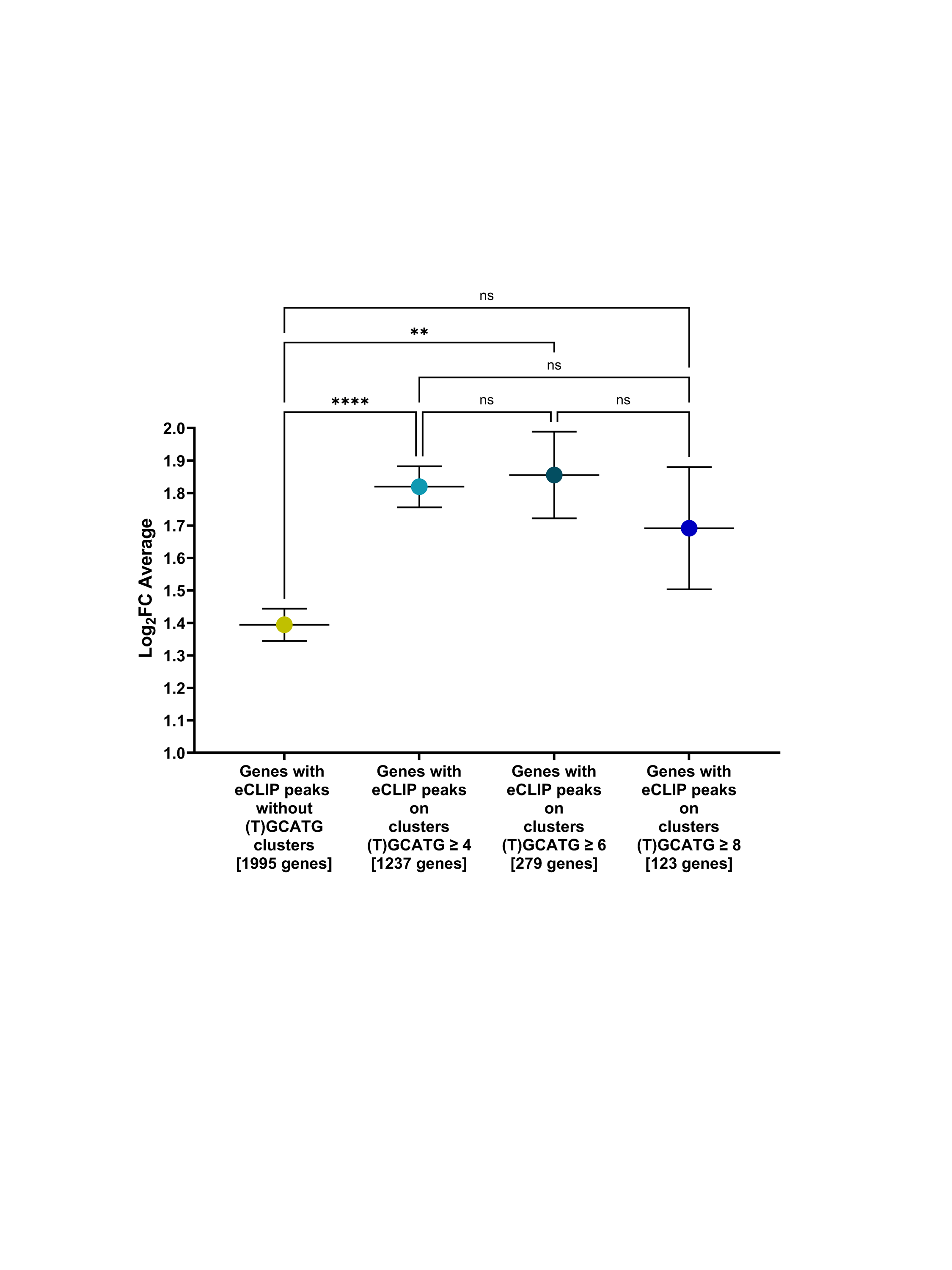

Supplement: S4 Fig — Graph showing the average of fold change isoform expression (Log2FC Average) for the set of genes with eCLIP peaks on clusters with different number of (T)GCATG motifs (≥ 4, 6 or 8) compared to genes with eCLIP peaks without (T)GCATG clusters. Note the dramatic inverse correlation in the number of genes with eCLIP peaks as clusters have increased (T)GCATG motifs number. ** p ≤ 0.01; *** p-value≤ 0.00001; ns, non-significant. (TIF) [file pgen.1011855.s004.tif]

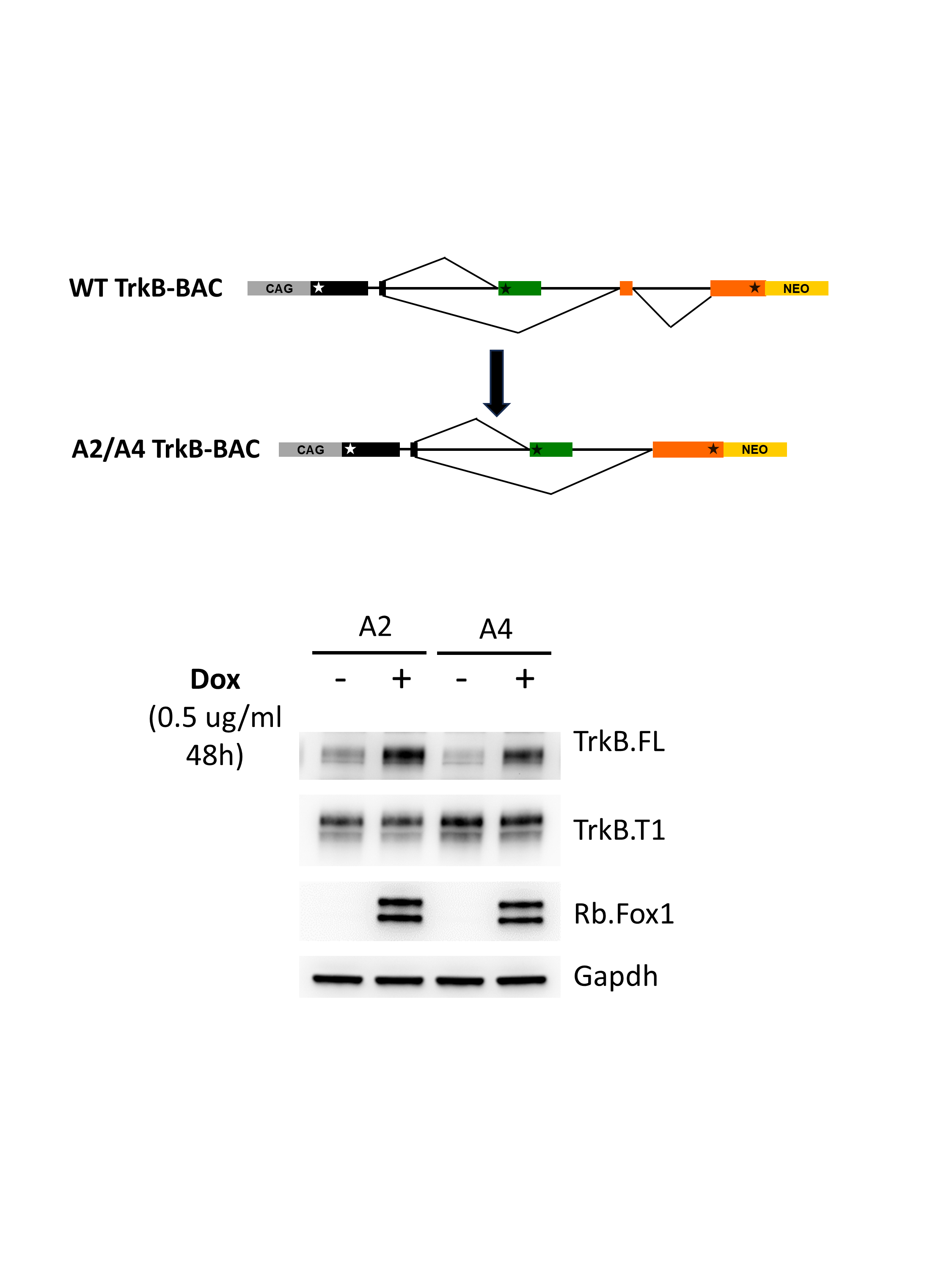

Supplement: S5 Fig — (A) Schematic representation of the WT TrkB-BAC and the TrkB-BACs with deletion of the intron immediately upstream of the TrkB kinase coding region. (B) Western blot analysis of TrkB.FL and TrkB.T1 protein expression levels from two independent cell lines (A2 and A4) with the TrkB-BAC minigene with the intron deletion, in the absence or presence of doxycycline (No Dox or +Dox 0.5 μg/ml for 48h); RbFox1- and Gapdh-specific antibodies were used, respectively, to verify doxycycline induction of Rbfox1 and as a control of protein loading. (TIF) [file pgen.1011855.s005.tif]

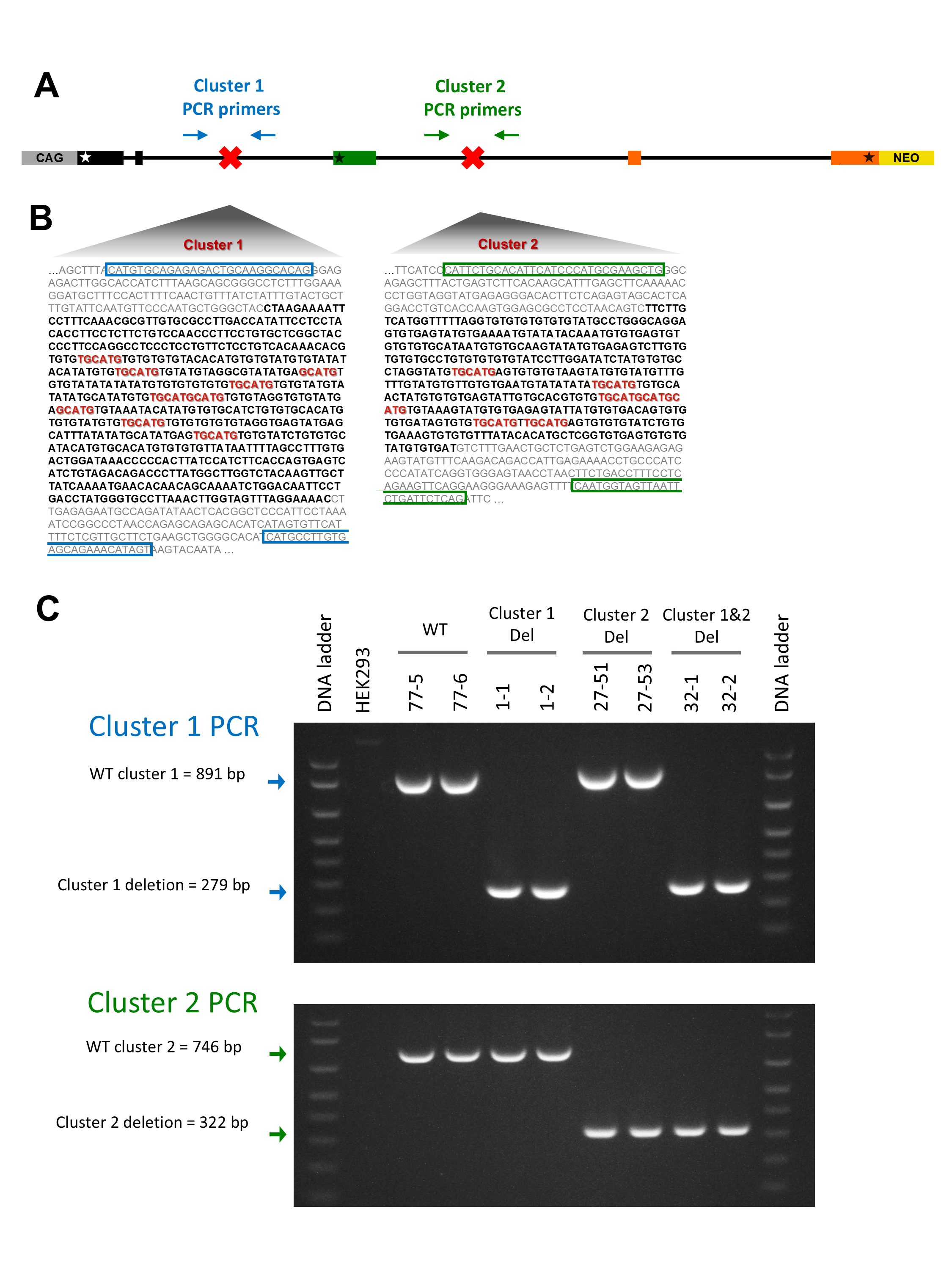

Supplement: S6 Fig — (A) Schematic representation of the TrkB-BAC minigene showing the position of the PCR-primers designed to detect the deletion of (T)GCATG-cluster 1 and (T)GCATG-cluster 2 (indicated by red X) analyzed in (C). (B) magnification of Cluster 1 and 2 areas indicating the location and sequence of the primers used for the analysis. (C) PCR analysis of genomic DNA from HEK293 cells used as negative control, cell lines expressing the ‘wild-type’ TrkB-BAC minigene (77−5 and 77−6 cells), cell lines expressing the TrkB-BAC minigene with cluster 1 deletion (1−1 and 1−2 cells), cell lines expressing the TrkB-BAC minigene with cluster 2 deletion (27−51 and 27−53 cells) and cell lines expressing the TrkB-BAC minigene with both cluster 1 and 2 deletion (32−1 and 32−2 cells). The PCR detecting cluster 1 deletion shows an amplicon of 891 bp (wild-type minigene sequence) and an amplicon of 279 bp (deletion of cluster 1). The PCR detecting cluster 2 deletion shows an amplicon of 746 bp (wild-type minigene sequence) and an amplicon of 322 bp (deletion of cluster 2). (TIF) [file pgen.1011855.s006.tif]

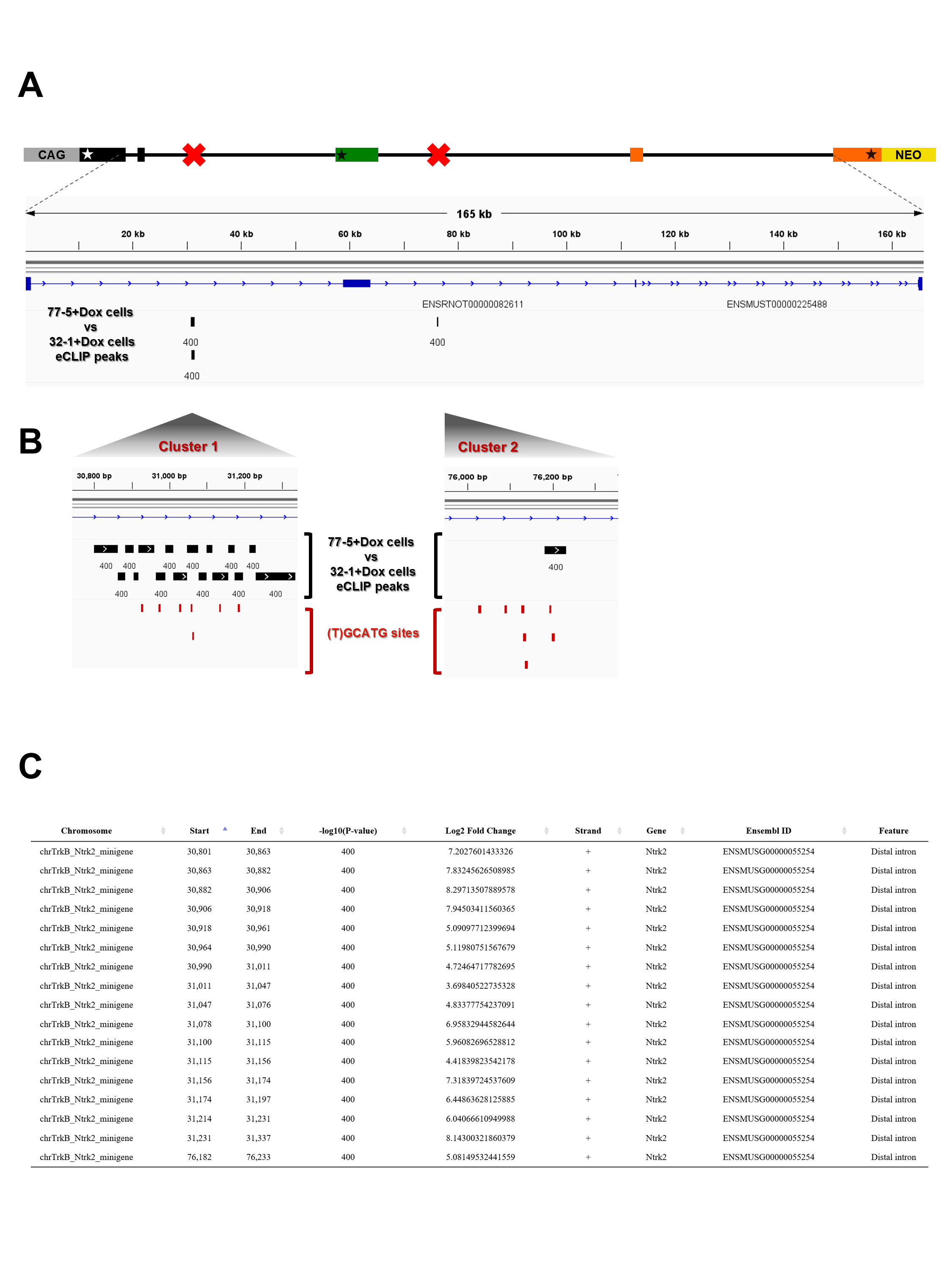

Supplement: S7 Fig — (A) Schematic representation of the TrkB-BAC minigene indicating the location of cluster1 and 2 deletion (red x) with the RbFox1 (+ Dox) eCLIP analysis of wild type TrkB-BAC expressing cells (line 77−5) compared to TrkB-BAC cells with cluster 1 and 2 deletions (line 32−1). Note the presence of only two statistically significant eCLIP areas in the intronic regions (black marks) corresponding to cluster 1 and 2. (B) Enlargement of the areas containing the two eCLIP peaks (indicated by shadowed arrows) corresponding to the (T)GCATG clusters (red marks). (C) Location, statistics, and fold change enrichment of all eCLIP peaks located in cluster 1 and cluster 2 in (B). (TIF) [file pgen.1011855.s007.tif]

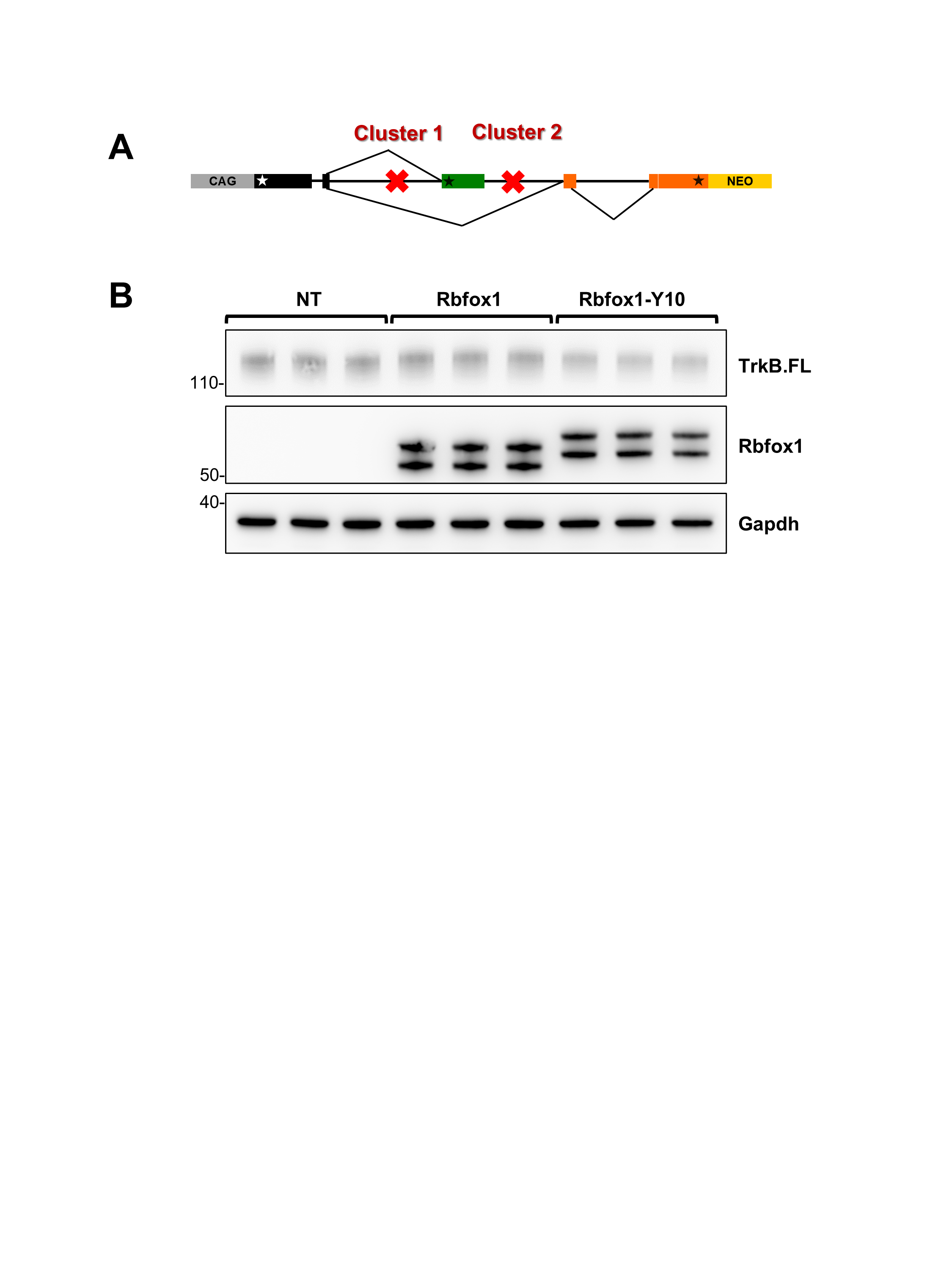

Supplement: S8 Fig — (A) Schematic representation of the TrkB-BAC minigene indicating the location of cluster1 and 2 deletion (red x). (B) Western blot analysis of lysates from HEK293 cells with the mutant cluster 1 and 2 deletion TrkB-BAC minigene 48h after transfection with a control (RbFox1) or an RbFox1 cDNA with mutations in 10 tyrosine residues in the CTD region [66] (Rbfox1-Y10). Non-transfected cells were used as control (NT). TrkB.FL, Rbfox1 and Gapdh protein levels were analyzed as in Fig 1. (TIF) [file pgen.1011855.s008.tif]
