# Supplementary material for: Clusters of deep intronic RbFox motifs embedded in large assembly of splicing regulators sequences regulate alternative splicing
Source: PLoS Genet. 2025 Sep 9;21(9):e1011855. doi: 10.1371/journal.pgen.1011855 (PMC12435732; doi:10.1371/journal.pgen.1011855)
Supplement: Attachment — Submitted filename: Response to Reviewers.docx [file pgen.1011855.s012.docx]

We want to sincerely thank the reviewers for their comments and suggestions. The comments are constructive and we thoroughly addressed them both experimentally and editorially to strengthen and improve our manuscript. Changes in the revised manuscript and responses to the reviewers below are in blue.

We hope that you will find our revised manuscript suitable for publication in Plos Genetics.

Responses to Reviewers’ Questions

**Reviewer #1:**

The manuscript by Tomassoni-Ardori et al. presents the discovery of two clusters of (U)GCAUG motifs flanking the TrkB.T1’s coding exons. These motifs are embedded within sequences bound by a large assembly of splicing regulators. Through bioinformatic analysis of multiple CLIP-seq and RNA-seq datasets, the authors propose that these deeply embedded clusters of the RbFox1-binding motif (U)GCAUG might be a crucial element in regulating the alternative splicing of numerous genes in the brain. They further conducted experiments and found that the deletion of these two clusters abolishes RbFox1's splicing regulatory function on TrkB, and the RbFox1-Y10 mutant still exhibits relatively lower splicing regulatory activity. This manuscript contributes to a better understanding of RbFox1's role in regulating TrkB expression. Overall, the findings are interesting; however, several issues need to be addressed.

We thank the reviewer for the positive assessment of our manuscript.

1. In Figs. 1 and 4, particularly in Fig. 4, how can the authors explain the multiple bands of western signals for both the FL and T1 proteins? Are these bands a result of other alternative splicing events occurring in the related region? To clarify this, RT-PCR results from the corresponding samples should be provided to reveal the details of alternative splicing in each assay.

The reviewer raises a valid point. We agree that we should have clarified the nature of the two bands, as we did in our original manuscript (Tomassoni-Ardori et al., eLife 2019, Figure 1A, right panel) where we used a specific TrkB.T1 antibody—now unfortunately unavailable—to demonstrate that the double band corresponds to the same TrkB.T1 isoform. That data suggested that the difference in molecular weight is due to glycosylation levels of TrkB.T1. However, since the current system is different we performed two additional experiments. First, we de-glycosylated the lysates from the two clones expressing the TrkB-BAC minigene (77-5 and 77-6). This showed only two bands: one for TrkB.FL and one for TrkB.T1. Second, as suggested by the reviewer, we performed RT-PCR and confirmed that only TrkB.FL and TrkB.T1 are significantly expressed. TrkB.T2, the second potential isoform generated by the BAC, is expressed at extremely low levels. We have added this information to the Results section (page 8, lines 157–160) and included the new data as Supplemental Figure 1.

2. In Fig. 2, how were the boundaries of the two clusters determined? Deleting such long regions could directly impact the binding of other splicing factors, as the authors mentioned regarding the LASR complex. Testing mutations of the (U)GCAUG motifs would be beneficial in addressing this concern.

We agree with the reviewer that mutating the (U)GCAUG motifs directly would be ideal. Unfortunately, these motifs are embedded within highly repetitive intronic sequences, which makes such an approach technically challenging. Recombineering or homologous recombination involving repetitive elements is extremely difficult and prone to off-target mutations. As a result, we opted to delete the entire sequences encompassing the (U)GCAUG clusters. While we acknowledge that this represents a limitation of our mutagenesis strategy, we would like to stress that, per se, the deletions are relatively small—612 bp for cluster 1 and 424 bp for cluster 2—especially in the context of the surrounding introns, which are approximately 50 kb in size.

Nonetheless, as the reviewer rightly points out, these regions are also enriched in repetitive elements associated with splicing regulation, such as TG/CA repeats and hnRNP motifs. Therefore, it is likely that deleting these sequences affects not only Rbfox1 binding but also that of other LASR complex members. However, we feel that the importance of the (U)GCAUG motifs for Rbfox1 activity is further supported by the complete loss of function observed when using an Rbfox1 mutant lacking RNA-binding ability (Fig. 1D–F).

3. What about the other five RbFox1-binding regions flanking the TrkB.T1-coding exons? If these regions were deleted, would similar effects on TrkB alternative splicing be expected? Conducting such tests could confirm the direct role of these clusters, independent of the effects of Rbfox1 abundance.

This is an interesting point, which we had considered during the course of the project. While the BAC-based system is a powerful tool that allows the study of splicing regulation in a large genetic context, it also involves a lengthy process, including BAC modification, transfection, and selection of individual cell clones for testing. This workflow typically takes 6–8 months, which has limited our ability to test all possible combinations of mutants.

Nevertheless, in one of our initial experiments, we generated a BAC mutant lacking the downstream 52 kb intron, which contains four of the eCLIP peaks shown in Figure 2A. Interestingly, as shown in the new panels of Suppl. Fig 2B, 2 of the eCLIP peaks have 2 and 3 RbFox1 binding sites and yet their deletions does not affect Rbfox1 regulation of TrkB isoform. These findings guided our focus toward the two ~50 kb introns flanking the TrkB.T1 coding exon and, most importantly, the eCLIP peaks corresponding to the two major (U)GCAUG clusters.

In response to the reviewer’s request regarding the relevance of the additional clusters, we have now included the new data (new Supplemental Figure 5 and Results page 13-13, line 256-261) showing that removal of four out of the seven eCLIP peaks is less impactful than removal of the two peaks associated with the clusters. Most importantly, we hope this new evidence further highlights two key conclusions of the paper: 1) The two deep intronic regions containing the (U)GCAUG clusters are essential for Rbfox1-mediated regulation of the BAC minigene; and 2) The remaining five Rbfox1-binding regions alone are insufficient to support Rbfox1-dependent modulation of TrkB isoform expression even if they have two or three RbFox binding sites.

4. In Figs. 4B and 4E, when one of the clusters is deleted, the expression of TrkB.FL increases. However, the level of TrkB.T1 either remains unchanged or even increases. How can this be explained?

In Fig 4B deletion of Cluster 1 shows no effect on RbFox1 regulation of TrkB isoforms since it is virtually identical to the pattern seen in Fig. 1C. In Fig 4E TrkB.FL increases while TrkB.T1 is unchanged. This suggest that this cluster is somewhat involved in other RbFox1 functions other than splicing. Although we did see something similar in our previous study (Tomassoni-Ardori 2019) we attributed this to an effect of RbFox1 on mRNA stability. Unfortunately, we have no idea of what the mechanism is. We hope that our BAC system may help shed light into future studies to address this point. Therefore, we have now added this point in the discussion section (page 18, line 379-387).

5. In Fig. 3C, the numbers 8,348 and 8,356 both incorporate the middle value 4,186. It would be more straightforward to subtract 4,186 from 8,348 and 8,356, respectively. Otherwise, it may confuse. The same problem exists in Fig. 3F.

We apologize for the confusion, and we thank all reviewers for pointing this out. We have now corrected both Venn diagrams in Fig. 3C and F following the reviewers’ suggestion, thank you (Page 10).

6. In line 187, the text reads "at least four or more (T)GCATG elements," while in line 273, it says "at least five or more (T)GCATG-motifs." Why are there two different criteria?

This is a mistake and we thank the reviewer for pointing this out. Indeed, our analysis was conducted using clusters with at least four or more (T)GCATG elements within 500 bp. We have now corrected the text.

**Reviewer #2**:

In their paper titled “Clusters of deep intronic RbFox motifs embedded in large assembly of splicing regulators sequences regulate alternative splicing,” Tomassoni-Ardori et al. identify clusters of Rbfox1 sites in large introns of TrkB that regulate the splicing of the TrkB.FL and TrkB.T1 isoforms. They construct a BAC minigene, and demonstrate that these sites are directly bound by Rbfox1, contain elements suggesting they are bound by LASR complex components, and when deleted alter the splicing pattern of TrkB. The authors further show that thousands of genes contain clusters of Rbfox1 sites in distal introns, and using available HITS-CLIP data show these clusters are indeed direct binding targets, and genes with bound Rbfox1 site clusters have higher levels of isoform expression diversity. The clusters are enriched in LASR-associated sequences, and the Rbfox1-Y10 mutant, previously shown to impair splicing activity and LASR formation, reduces levels of reporter expression.

The data on TrkB regulation by Rbfox1 is straight-forward and convincing. The BAC minigene is an elegant tool, and has a strong potential for use in future studies to understand Rbfox1 and LASR function. The paper is an important contribution to the field, and will be of interest to the splicing and Rbfox1 fields. The data on CTD function and LASR involvement is less convincing, and the text should be modified to reflect alternate interpretations of these results. I have the following suggestions to improve the manuscript:

We thank the reviewer for the positive comments on our manuscript and constructive suggestions below.

The number of genes with peaks in Figure 3C and discussed in the text (Lines 190-Line 193) do not agree. The authors should use the actual counts (8348 and 8356) rather than “about 8300”.

We thank also this reviewer for the comment which, as noted for reviewer 1, we have addressed in page 10.

In addition, this Venn Diagram seems to be incorrectly labeled, as it would suggest that there are 8356 + 4186 or 12542 genes with HITS-CLIP peaks in the Weyn-Vanhentenryck data and 8348 + 4186 or 12534 genes in the mouse genome. The Venn Diagram in Figure 3F seems to have the same issue. The total number of genes in the circle should be moved outside of the circle, or it looks like it denotes the number of genes in a specific region of the circle.

We thank the reviewers for pointing out our mistake. We have now changed the Venn diagrams in both Fig. 3C and 3F following the reviewers’ suggestion.

Can the authors comment on the number of Rbfox1 binding sites required in a “cluster” region to retain the effects they report here? They identify clusters as more than 5 (T)GCATG sites, but what is the range of binding sites in these regions? Are clusters with for example >10 sites common and more effective?

Within the vicinity of the other eCLIP peaks in TrkB, how many (T)GCATG sites are present?

Are the clustered Rbfox1 sites the key element to these clusters, or would they still be function with 1-2 Rbfox1 sites? In other words, are Rbfox1 site clusters the defining feature of these elements, or do the concentration of other LASR binding elements play a role?

The reviewer raises several interesting issues.

To address the question of whether the number of RbFox binding sites within a cluster enhances its regulatory effect, we extended our analysis beyond the initial threshold of four binding sites. Specifically, we included additional analyses using stricter cut-offs—clusters containing more than six and eight (T)GCATG sites. Interestingly, we observed that the average isoform expression change (Log2FC Average) for genes with eCLIP peaks overlapping (T)GCATG clusters remained consistent, regardless of the number of binding sites within the cluster. Moreover, the number of genes with clusters meeting these higher thresholds decreased sharply as the number of (T)GCATG sites increased: 1237 genes with clusters having ≥4 sites, 279 with ≥6 sites, and only 123 with ≥8 sites. These findings indicate that genes containing clusters with a high number of (T)GCATG motifs are relatively rare. Taken together, our analysis suggests that increasing the number of RbFox binding sites within a cluster is not a major determinant of RbFox-mediated splicing regulation. These data have been added as a new Suppl. Fig 4 (Page 12, line 239-251)

To address the question regarding the number of (T)GCATG sites near other eCLIP peaks, we have now included data on the additional five eCLIP peaks identified in the TrkB-BAC minigene system (Supplementary Figure 2, panels A and B) and have amended the Results section accordingly. Notably, one of these five peaks is located near a region containing three (T)GCATG motifs (Supplementary Figure 2B). However, deletion of the intron harboring this motif—along with three other eCLIP peaks—does not impact RbFox1-mediated TrkB.FL expression (new Suppl Fig 5). Importantly, the region containing these four (T)GCATG sites lacks repetitive sequences typically associated with LASR binding, suggesting that sequence context, in addition to motif number, plays a critical role.

Lastly to further expand on our knowledge of cluster of (U)GCAUG motifs on RbFox protein function on splicing, we took the liberty to perform additional analyses using RbFox2 and RbFox3, which share the same RNA recognition motif (RRM) as RbFox1. Interestingly, both RbFox2 and RbFox3 regulate TrkB isoform expression in a manner similar to RbFox1, and their regulatory effects are abolished by mutations in Cluster 1 and 2. However, the magnitude of TrkB.FL splicing modulation is reduced, particularly for RbFox3 (see new Figure 5). These findings support a degree of functional redundancy among RbFox family members, as previously reported, and point to the importance of the (U)GCAUG motifs as a critical component of the clusters. The new data have been incorporated into the Results section as Figure 5 (results page 14) and discussed on page 21 (line 446-449) in the context of the utility of our system to screen for functionality of human RbFox2 and 3 mutants.

The authors overstate the outcome of their experiments with Rbfox1-Y10. The authors infer details on the assembly of LASR based on use of Rbfox1-Y10, but they do not test assembly of LASR directly. Rbfox1-Y10 splicing levels of TrkB are indeed significantly reduced in Figure 5E,F. In lines 303-304 the authors claim the data suggest the CTD requirement to mediate higher-order assembly of LASR for splicing activation by Rbfox is not absolute. This interpretation may not be correct, as Ying et al., 2017 show that the Y10 mutant can mediate some splice events (see in particular Figure 5A and 4D in that paper) and that region C3 of the CTD can interact (albeit weakly) with LASR. It is therefore not clear that the Rbfox1-Y10 should abrogate interaction with LASR or completely inhibit splicing. The authors’ data is consistent with a strongly reduced function of Rbfox1, but the CTD likely still has some functionality with Rbfox1-Y10.

This point is well taken as we have not tested experimentally the LASR complex assembly and, as suggested by the reviewer, some weak interaction of the RbFox1-Y10 is still possible with the LASR. As such, we have removed the sentence with that conclusion and modified the text in the abstract, results and discussion sections.

The authors need to add citations to relevant regions of the discussion, for example lines 312-315. These concepts seem to be from the literature, but citations are missing. In addition, the authors could expand their discussion of RNA-looping, which is a very intriguing point suggested by their results, but they only cite a single paper (Lovci et al, 2013). In addition, the authors could provide another example or two of long-intron containing genes where the long introns are critical to splicing regulation, as the only long-intron containing example provided is TrkB.T1 (lines 334-338), which is the topic of the paper.

We appreciate the reviewer’s comment and have noted the deficiencies. We have now included additional references in the appropriate sections (e.g. page 16-17 line 345-346, 349) and expanded the part on RNA looping including the utility of the CRIC-seq to study RNA RbFox1 mediated RNA looping in splicing regulation (page 20, line 429-434). Lastly, we have included other examples of long-intron containing genes where the long introns are critical to splicing regulation (page 18, 19, line 391-398).

The Methods section needs additional details. Line 403. In the methods, the length of Dox treatment before harvest should be included. What confluency were the cells grow to before Dox treatment? The confluency and length of transfection also should be included in line 428. Line 407. Were the cells lysed directly in the cell culture dish? Lines 416-420. What concentrations were the primary and secondary antibodies used at? Line 433. What cDNA generated using oligo-dT or random primers? Were there any modifications to the SSIII protocol?

We thank the reviewer for requesting these missing details which will help the reader with the methodology. We have now revised the methods section and included all these details (highlighted in page 22 and 23).

**Reviewer #3**:

The authors address the function of deep intron splicing regulatory elements, an under-studied phenomenon that likely plays a critical role in splicing of many genes. They use BAC-based minigenes, an interesting approach to identify and explore the function of new elements in deep distal intron regions. The TrkB gene was used as the model for study,

The data clearly show that deep intron clusters of Rbfox binding sites flanking the terminal T1 exon strongly influence the balance of T1 vs FL isoforms in RNA expressed from the BAC minigene. Moreover, an Rbfox1 mutation that interferes with binding was able to abrogate this effect on splicing. These results certainly support the general hypothesis that deep intron sequences can modulate splicing of exons mapping tens of kb distant from the regulatory motifs.

We thank the reviewer for appreciating our study.

However, there is concern about whether the specific BAC minigene used in this study accurately represents the in vivo situation. One might expect the first experiment would be to test whether the minigene behaves like the endogenous gene with respect to splicing regulation by Rbfox1. But the authors’ 2019 paper in Elife reported that “Rbfox1 upregulation increases TrkB.T1 expression in vivo”, relative to TrkB.FL. In contrast, the current manuscript observes the converse, an increase in TrkB.FL isoform expression associated with over-expression of Rbfox1. Is there a logical explanation for the disparity that would justify the use of this model?

The reviewer is correct. Initially we developed this model system to identify elements in the introns surrounding the TrkB.T1coding exon that contribute to the regulation of expression of this isoform. Although we modified the BAC to express both TrkB isoforms, it does not include all intronic regions, limiting its ability to fully recapitulate the complete TrkB gene physiological regulation. For instance, a distal intronic region in the kinase domain contains 15 clustered (U)GCAUG motifs in highly repetitive sequences (Supplementary Table 1), which may regulate TrkB isoforms in vivo but is absent from our construct. In addition, in this study, Rbfox1 is expressed in human HEK293 cells, which lack endogenous Rbfox1, whereas our prior in vivo work used neuronal cells with native Rbfox1 expression. The splicing environment likely differs between human HEK293 and murine neurons. Despite these limitations, we feel that the TrkB-BAC minigene proved valuable for identifying (U)GCAUG clusters in deep intronic regions and highlights the utility of BAC-based systems for dissecting alternative splicing mechanisms. This approach may offer additional advantages in the study of deep intronic investigations particularly for genes that fall within the BAC size limits. We have now included this important point in the revised manuscript discussion (page 17-18, lines 362-378).

1. The authors’ bioinformatic analyses demonstrate clearly that (U)GCAUG clusters are widely present in both mouse and human genes. Further, they show that genes with deep intron (T)GCATG clusters mapping to Rbfox1-eCLIP sites are more likely to be alternatively spliced in an Rbfox1-dependent manner than genes lacking this Rbfox1 cluster/eCLIP overlap. These results certainly support the main hypothesis that deep intron (T)GCATG clusters are important regulators of alternative splicing.

We thank the reviewer for emphasizing our conclusion and for appreciating our study.

2. The authors also show that these (T)GCATG clusters are enriched for motifs known to bind other components of the LASR complex, supporting the model in which Rbfox1 functions in the context of LASR complexes. However, the authors may want to temper the conclusion that “clustered (U)GCAUG-motifs promote the recruitment of RbFox1 proteins to form a Rbfox1/LASR complex required for splicing” (from the abstract). While this is a reasonable hypothesis, they haven’t actually demonstrated the presence of LASR complexes.

The point is well taken and on suggestion of the reviewer we have temper down the indicated sentence by making it a more hypothetical conclusion (“Our data raise the possibility that…” Abstract, line 38).

3. Line 219: More information is needed to evaluate the significance of the statement that “we found that about 40% of the genes involved in these pathologies have (T)GCATG-clusters including brain disease 36.5%, muscle disorders 40.8% and heart disease 39.5%” ? Are these percentages higher than the percent of all expressed genes in these tissues having (T)GCATG clusters? If 9826 human genes have (T)GCATG clusters in all, is this close to 40% of all genes?

Our intent was to merely provide a list of (T)GCATG-clusters present in genes associated with diseases in tissues where Rbfox1 is predominantly expressed (brain, heart and skeletal muscles). However, following the reviewer’s comment we have decided to remove this sentence.

4. Figure 3C, F. Other reviewers can correct me if I’m wrong, but I think the numbers in the Venn diagrams are not presented in the conventional manner. I think the labels outside of the shared regions should represent the numbers of unshared events, not the total events in the encompassing circle.

Again, we thank the reviewer for the suggestion and apologize for the confusion. As noted in response to the same comment by the other reviewers we have fixed the Venn diagrams in Fig. 3.

5. Figure 3H. I don’t understand the figure due to some confusion about the axes. The X-axis is labeled as fold change; what does 100% fold change mean? I think a two-fold change in isoform expression would be a 100% increase, correct? Are there no examples of a greater than two-fold change in isoform expression? Can I interpret the graph to mean that ~25% of genes with (T)GCATG clusters on eCLIP peaks show a 100% (2-fold) change in isoform expression? If I’m interpreting this correctly, a change in isoform expression from 0.5% to 4% would be more significant (higher fold change) than a change from 20% to 80%, but one would think the latter might be more biologically significant.

We apologize if Figure 3H generated some confusion and note that the interpretation of the reviewer is correct. We have now modified the X axis of panel H to include “Expression change (%)” to clarify that it is not a fold change. Also, to answer to the reviewer about whether there are “examples of a greater than two-fold change in isoform expression”, the answer is yes, but the number is low as indicated by the gradual decrease in the Y axis of the number of genes with an increase in gene isoform expression as we reached the 100%.

6. Figure 6. Since proteins are generally presented with N-termini at the left and C-termini at the right, the diagrams in Figure 6 seem backwards (the tyrosine should be C-terminal relative to the RRM). This could be clarified by labeling the N- and C-termini.

We agree with the reviewer’s comment. Following the suggestion, we have now added the N- and C-terminal in the schematic in the current Figure 7.

7. The 2023 paper by Li et al., in JCI, does not have the complete citation.

We thank the reviewer for noticing the omission due to a problem with this citation in endnote. The citation has been completed in the revised manuscript.
